# GWAS for Starch-Related Parameters in *Japonica* Rice (*Oryza sativa* L.)

**DOI:** 10.3390/plants8080292

**Published:** 2019-08-19

**Authors:** Chiara Biselli, Andrea Volante, Francesca Desiderio, Alessandro Tondelli, Alberto Gianinetti, Franca Finocchiaro, Federica Taddei, Laura Gazza, Daniela Sgrulletta, Luigi Cattivelli, Giampiero Valè

**Affiliations:** 1 Council for Agricultural Research and Economics, Research Centre for Genomics and Bioinformatics, via S. Protaso 302, 29017 Fiorenzuola d’Arda (PC), Italy; 2 Council for Agricultural Research and Economics, Research Centre for Forestry and Wood, viale Santa Margherita 80, 52100 Arezzo, Italy; 3 Council for Agricultural Research and Economics, Research Centre for Cereal and Industrial Crops, s.s. 11 to Torino, km 2.5, 13100 Vercelli, Italy; 4 Council for Agricultural Research and Economics, Research Centre for Engineering and Agro-Food Processing, Via Manziana 30, 00189 Roma, Italy; 5 Dipartimento di Scienze e Innovazione Tecnologica, Complesso Universitario S. Giuseppe, University of Piemonte Orientale, Piazza S. Eusebio 5, 13100 Vercelli, Italy

**Keywords:** resistant starch, apparent amylose content, marker-trait associations, GWAS, rice quality

## Abstract

Rice quality is mainly related to the following two starch components, apparent amylose content (AAC) and resistant starch (RS). The former affects grain cooking properties, while RS acts as a prebiotic. In the present study, a Genome Wide Association Scan (GWAS) was performed using 115 rice *japonica* accessions, including tropical and temperate genotypes, with the purpose of expanding the knowledge of the genetic bases affecting RS and AAC. High phenotypic variation was recorded for the two traits, which positively correlated. Moreover, both the parameters correlated with seed length (positive correlation) and seed width (negative correlation). A correlational selection according to human preferences has been hypothesized for the two starch traits and grain size. In addition, human selection has been proposed as the causal agent even for the different phenotypes related to starch and grain size showed by the tropical and temperate *japonica* accessions utilized in this study. The present GWAS led to the identification of 11 associations for RS on seven chromosomes and five associations for AAC on chromosome 6. Candidate genes and co-positional relationships with quantitative trait loci (QTLs) previously identified as affecting RS and AAC were identified for 6 associations. The candidate genes and the new RS- and/or AAC-associated regions detected provide valuable sources for future functional characterizations and for breeding programs aimed at improving rice grain quality.

## 1. Introduction

Rice provides more than 1/5 of calories for human worldwide populations (http://faostat.fao.org) and, in the last decades, the economic development and the improvement of lifestyle has brought more concerns about rice quality. Rice quality is primarily related to the composition of the main component of grains, starch. The percentage of amylose on total starch, referred as apparent amylose content (AAC), is the first factor affecting the cooking properties of rice grains [1]. Based on AAC, rice genotypes have been classified as waxy (0–5%), low (5–20%), medium (20–25%), and high (26–33%) [2,3]. After cooking, the grains of high AAC rice varieties remain firm and separate, whereas low AAC genotypes result in tender, glossy, and cohesive grains [1]. AAC in rice grains is directly associated with the amounts of the granule bound starch synthase I (GBSSI) enzyme, which regulates starch accumulation in the endosperm of developing caryopsis [4]. This enzyme is encoded by the *Waxy* (*Wx*) gene and, as extensively reported, different alleles at this locus correspond to different AAC percentages [1,4,5,6,7]. The low and high AAC classes are mainly discriminated by the presence, respectively, of two different wild type alleles at the *Wx* locus, as follows: *Wx^a^*, associated with high AAC levels and *Wx^b^*, carried by low AAC rice cultivars [8]. The presence of the two alleles is the result of a G to T SNP (single nucleotide polymorphism) at the 5′ splice site of the *Wx* intron 1, which causes an alternative splicing, thus reducing the production of functional enzymes in rice endosperm and determining the occurrence of low AAC amounts [1,4,5,6,7]. Another *Wx* allele associated with AAC variation is represented by *Wx^in^*, frequently in accessions belonging to an aromatic group and to tropical *japonica* exhibiting an intermediate AAC [8]. This allele is generated by an A to C non-conservative mutation in *Wx* exon 6, which determines a Tyr to Ser substitution in the active site of the enzyme, reducing the specificity for its substrate [8].

The increase of metabolic diseases related to erratic dietary habits, like diabetes, hypertension, and obesity, has oriented the recent breeding programs to improve the content of bioactive compounds in consumed food. Resistant starch (RS) is a dietary fiber representing the part of starch that is not digestible by the enzymes of the stomach and the small intestine, but remains intact until reaching the colon where it acts as a prebiotic [9]. The beneficial effects of RS on health consist in the improvement of insulin sensitivity [10], the decreasing of postprandial blood glucose [11,12,13,14], and the prevention of colon rectal cancer [14,15]. Moreover, RS may assist in the prevention and management of conditions associated with the metabolic syndrome [16] and could act as a satiety agent [17]. RS is present in different plants, such as bananas, potatoes, and cereals, and its content is genetically determined even if it is also affected by the thermal treatment applied during food processing [18,19].

There is a wide genetic diversity for RS in rice. RS levels vary from 1.2% to 9.0% of cooked polished rice dry weight in commercial normal-starch rice varieties, across different growing locations [20]. Evaluations carried out using brown uncooked rice [21] suggested that *indica* varieties have higher levels of RS (from 0.1 to 3.2%) with respect to *japonica* varieties (from 0 to 1.6%). In addition, in rice, as in other cereals, RS is affected by starch composition; for example, it positively correlates with AAC [22,23,24]. More specifically, elevation in RS is traditionally associated with the activity of enzymes that affect starch composition, as follows: GBSSI positively controls the amount of RS [25], whereas the activity of the starch branching enzyme IIb (SBEIIb) has a negative effect on the level of RS. In the latter case, down-regulation and mutation of the SBEIIb-encoding gene causes an increase of long chain amylopectin, which mimics the role of amylose in conferring elevated AAC and RS [26]. In fact, the *amylose extender* mutant IR36*ae*, exhibiting an inactive SBEIIb that results in alterations in the amylopectin fine structure, has a higher level of RS in cooked grains with respect to the wild type [26]. A higher percentage of RS, in comparison to the wild type, was also found in Goami 2 (a mutant of cultivar Ilpumbyeo), which carries a mutation mimicking the amylose extender phenotype [27]. A negative role of SBEIIb in affecting RS content has also been demonstrated in wheat [28].

Since for most rice cultivars the RS content is generally under 3% in milled grains, which is not enough to confer the associated health benefits [21,29], many studies have been focused on the elevation of RS content using mutation breeding and bioengineering [29,30]. Such studies have revealed an important role of SBEs, the critical enzyme for the formation of amylopectin branches [31], in determining the RS content. As an example, the high AAC transgenic rice line Teqing Resistant Starch, modified by antisense RNA for inhibition of SBEI and SBEIII, yielded substantially higher RS, with respect to its wild-type genotype [22,32]. The down-regulation of SBEIIb in rice endosperm by artificial microRNA (*amiRNA*) was associated with increased AAC, with only modest changes in the total starch (TS) content and with an increase in the proportion of long and intermediate amylopectin chains [26]. This led to severe alterations in starch granule morphology and crystallinity, as well as elevated levels of RS and lower values of the glycaemic index [26]. In accordance, the amount of RS in gelatinized starch showed an overall negative correlation with the amylopectin short branch-chain content and with the average chain length of amylopectin when data from starches of the rice cultivar Teqing and its high AAC transgenic line Teqing resistant starch, as well as from normal and high-amylose maize starches, were analyzed [33]. Correspondingly, a high degree of amylolysis was related to a high proportion of short amylopectin branch chains in amylopectin isolated from wheat, triticale, corn, and barley starches [34].

The importance of the *SBE* loci in determining RS content in rice was confirmed by the mapping of *sbe3-rs*, a quantitative trait locus (QTL) for RS on rice chromosome 2, using a mapping population obtained from the cross between the high RS *japonica* mutant Jiangtangdao 1 and the *indica* cultivar Miyang 23 [29]. In this work, *SBE3*, which encodes for a SBE, was suggested as the candidate gene for the QTL region [29].

In addition to SBEs, other enzymes implicated in starch metabolism have been demonstrated to affect RS in rice. High RS rice mutants, indeed, displayed lower activity of SBEs and ADP-glucose pyrosphorylase (AGPase), catalyzing the first rate-limiting reaction of starch biosynthesis, and higher activities of starch synthases (SSs) and starch de-branching enzymes (DBEs), with both affecting the number of short-chain amylopectins [35]. A map-based cloning of a RS locus in *indica* rice [36] identified a defective *ssIIIa* allele, resulting from the introduction of a premature stop codon, which was responsible for an increased RS production. Furthermore, it was shown that the high RS phenotype was also dependent on the high expression of the *Wx^a^* allele [36].

Despite the evidence described above, to date, limited information is available on the genetic bases controlling the variability of RS in rice. By segregation analysis of a population using the RS111 mutant as the common high-RS parent, SSR markers on chromosomes 8 and 6 were found to be associated with RS in two F_2_ populations [37]. Moreover, two QTLs for RS have been mapped on chromosome 7 (*qRS7-1* and *qRS7-2*) in brown and polish rice, using a F_2–3_ family derived from the cross between the two rice genotypes Gongmi No. 3 and Diantun 502 [38]. More recently, a Genome Wide Association Scan (GWAS) analysis on 105 rice accessions, including *japonica* and *indica* genotypes, led to the discovery of four RS-related QTLs, explaining from 10% to 13% of total phenotypic variation. Two QTLs were located on chromosome 6 and corresponded to the *Wx* and the *SSIIa* loci, respectively, while the other two QTLs were localized to chromosomes 8 and 9, proximal to the SBEI and ADPase small subunit I encoding loci, respectively [24]. In barley, a lesion of the *SSIIa* gene results into a shortened amylopectin chain length distribution and negative pleiotropic effects on SBEIIa and SBEIIb activity [39], thereby arguably affecting the RS level.

In the present study, a GWAS approach was performed on a *japonica* rice population, including 115 accessions genotyped by the genotyping by sequencing (GBS) method, with the aim of expanding the understanding about the genetic basis of the determination of RS content and AAC.

## 2. Results

### 2.1. Assessment of Starch-Related Parameters in the Japonica Rice Population

Non-cooked brown rice of the 115 rice genotypes were analyzed for TS and RS content. On average, the TS content was 73.43 ± 1.69%, with Graal showing the lowest value (70.40%) and Lagrue the highest one (81.73%; Table S1). RS content ranged from 0.006% (Miara) to 0.326% (Escarlate; Table S1), with a mean value of 0.1 ± 0.066%. Data were also reported as RSTS ratio percentages, which allowed the quantification of RS content based on the corresponding TS value. The RSTS ratio ranged from 0.008% (Miara) to 0.44% (Escarlate; Supplementary Table S1), with a mean value of 0.132 ± 0.088%.

**Figure 1:**
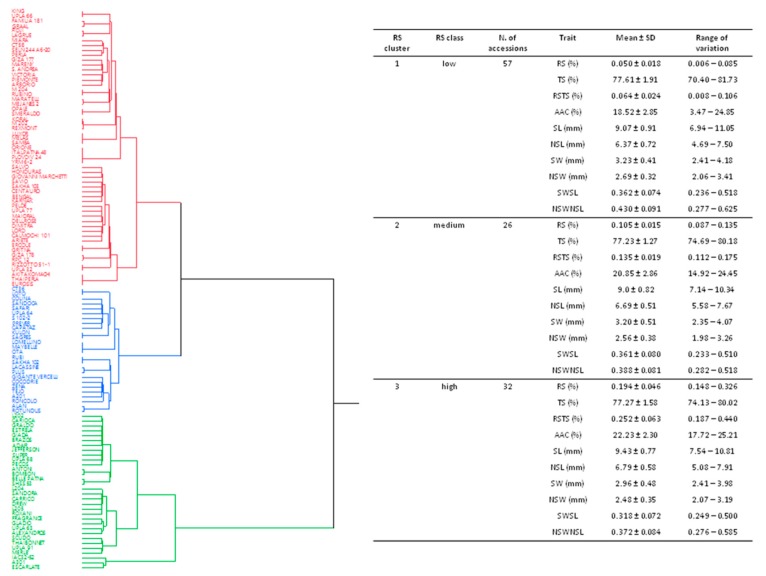
Hierarchical clustering based on resistant starch (RS) and RS over total starch (RSTS) values using Euclidean distances (graph) and the summary of the mean values and the ranges of variation of each phenotypic trait recorded for the different RS clusters (table). Each color corresponds to a specific RS cluster, as follows: Red = RS cluster 1; blue = RS cluster 2; green = RS cluster 3. SD = standard deviation; RS = resistant starch; TS = total starch; RSTS = ratio between RS and TS; AAC = apparent amylose content; SL = seed length; SW = seed width; NSL = naked seed length; NSW = naked seed width; SWSL = ratio between SW and SL; NSWNSL = ratio between NSW and NSL.

A hierarchical clustering, based on the two variables, RS and RSTS, and using Euclidean distances, separated the 115 rice genotypes into three main clusters (Figure 1), as follows: Low RS, constituted by the 57 accessions belonging to cluster 1 (RS from 0.006 to 0.085%; RSTS between 0.008% and 0.106%); medium RS, including the 26 genotypes belonging to cluster 2 (RS from 0.087 to 0.135%; RSTS from 0.112 to 0.175%); high RS, containing 32 accessions belonging to cluster 3 (RS from 0.148 to 0.326%; RSTS between 0.187% and 0.440%; Figure 1 and Supplementary Table S1).

The AAC values were evaluated in a previous work [1] on the seeds of the 115 accessions and re-analyzed in the present work to study the relation between AAC and RS. The AAC values ranged from 3.47% (for the waxy cultivar Calmochi 101) to 25.21% (L202), with an average of 20.08 ± 3.14% (Supplementary Table S1). According to the commercial rice classification [3], the 115 genotypes were classified into four AAC classes, as follows: Low-waxy, corresponding to Calmochi 101 (ACC = 3.47%); low, including 66 genotypes showing AAC values ranging from 14.92 to 19.95% (average 18.17 ± 1.20%); medium, with 46 accessions displaying AAC values from 20.05 to 24.85% (average 22.95 ± 1.36%); high, including the two genotypes Fragrance (25.16%) and L202 (25.21%; Supplementary Table S1).

**Figure 2:**
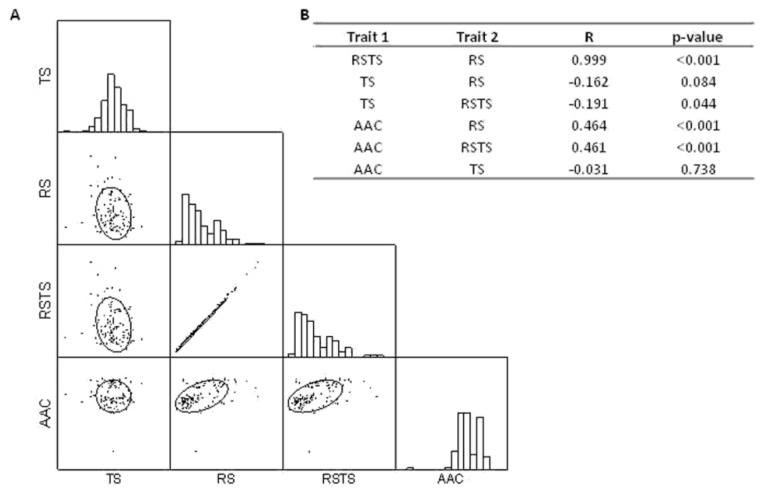
Correlation between the starch-related traits recorded in the present study. (**A**) Scatterplot matrix of starch-related traits. Histogram distribution plots for the single variables are shown on the diagonal cells. Below, the bivariate distributions are reported for each pair of traits. Confidence ellipses mark the confidence limit for each distribution (*p* = 0.95). (**B**) Pearson’s correlation coefficients (R) for each pair of traits (Trait 1 and Trait 2); *p*-values are also reported. RS = resistant starch; TS = total starch; RSTS = ratio between RS and TS; AAC = apparent amylose content.

Pairwise Pearson’s coefficients of correlation among traits were calculated and highly significant (*p* < 0.001) positive correlations were detected between RS and RSTS (*R* = 0.999), RS and AAC (*R* = 0.464), and RSTS and AAC (*R* = 0.461; Figure 2).

In agreement with the positive correlation between RS and AAC, significant differences of AAC were recorded between the three defined RS groups. The AAC mean value observed for the high RS cluster 3 (22.23 ± 2.30%) was higher with respect to the other clusters, while the lowest AAC mean value (18.52 ± 2.85%) was recorded for cluster 1, corresponding to the low RS class (Figure 1). The Wilcoxon test, conducted considering the mean values of each trait calculated for each RS cluster, revealed significantly different AAC levels, mainly between RS clusters 1 and 3 (Z = 4.170; Supplementary Table S2). No significant correlation was found between TS and the other starch-related traits (Figure 2).

**Figure 3:**
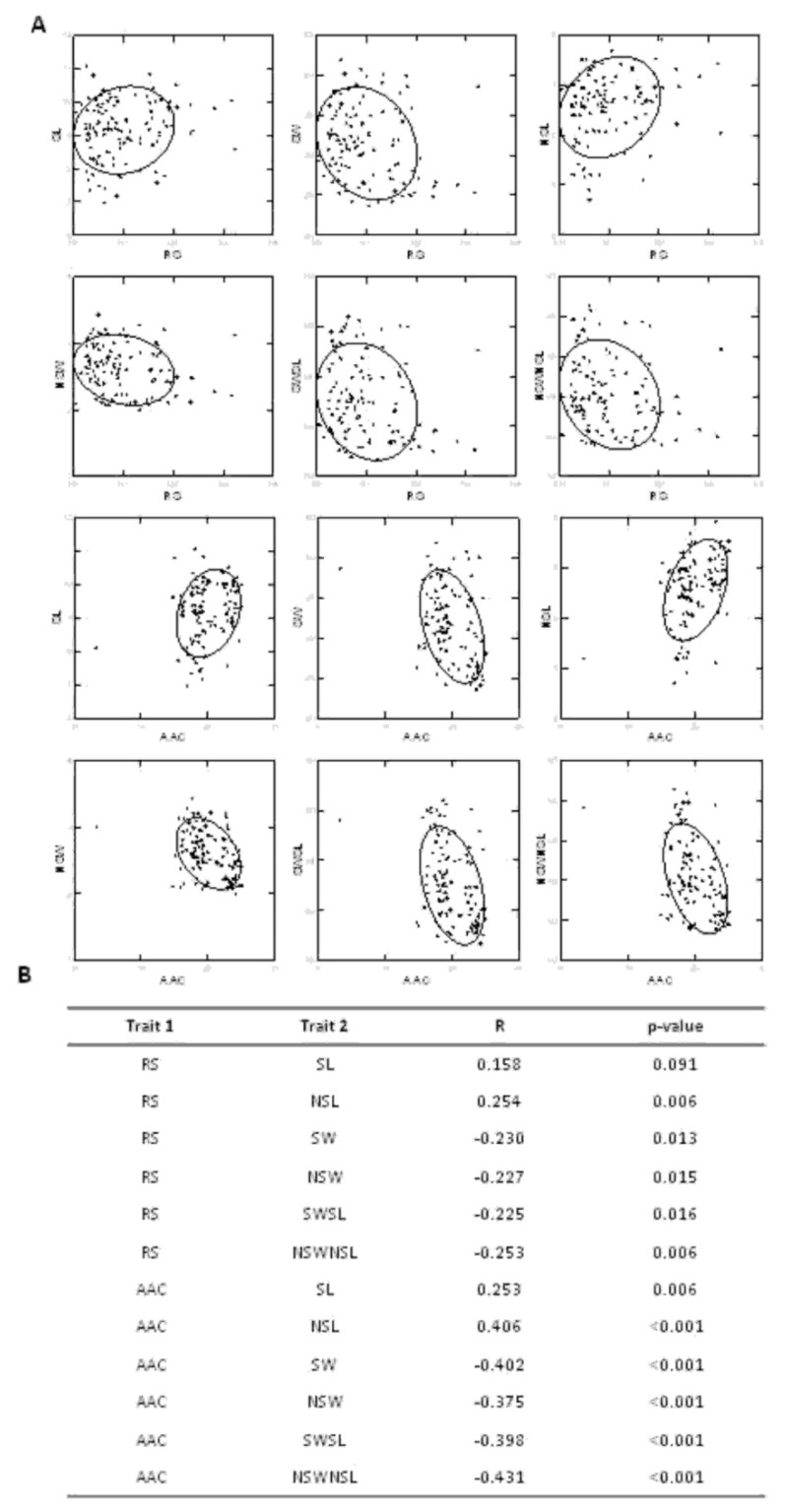
Correlation between resistant starch (RS) or apparent amylose content (AAC) and grain shape-related traits. (**A**) Scatterplots of RS or AAC and grain shape-related traits, representing the bivariate distribution for each pair of traits. Confidence ellipses mark the confidence limit for each distribution (*p* = 0.95). (**B**) Pearson’s correlation coefficients (R) for each pair of traits (Trait 1 and Trait 2); *p*-values are also reported. Probabilities were adjusted by Bonferroni’s correction for multiple tests. RS = resistant starch; AAC = apparent amylose content; SL = seed length; SW = seed width; NSL = naked seed length; NSW = naked seed width; SWSL = ratio between SW and SL; NSWNSL = ratio between NSW and NSL.

According to previous results [1], AAC correlated positively with seed length, mainly for naked seeds, while negative correlations were found between AAC and SW, NSW, SWSL, and NSWNSL (Figure 3).

Significant differences were recorded for some grain shape related traits calculated for the low and medium AAC classes, particularly for SWSL and NSWNSL, for which higher values were calculated for the low AAC class, with respect to the medium one (Tables S3 and S4).

The same type of correlation as for AAC was found between RS (and RSTS) and the grain shape-related traits, even considering the lower *p*-values (Figure 3 and Supplementary Figure S1). Accordingly, RS clusters 1 and 2 (low and medium RS, respectively) displayed higher SW, NSW, SWSL, and NSWNSL mean values, which negatively correlated with RS, with respect to the high RS cluster 3 (Figure 3; Supplementary Table S2).

No significant correlations were recorded between TS and the traits related to grain shape (Supplementary Figure S1).

Considering our rice panel, significant higher mean values of RS, RSTS, and AAC were found in tropical *japonica* accessions (45 accessions) with respect to the temperate ones (70 accessions) (Supplementary Table S5), while no differences were observed for TS. Differences among temperate and tropical *japonica* genotypes were also recorded for all the traits related to grain shape. The tropical varieties showed longer and thinner grains, with lower SWSL and NSWNSL values, with respect to the temperate ones (Supplementary Table S5).

The ANOVA analysis revealed significant genotypic variability for RS and AAC, with no substantial effect of replicates (Supplementary Table S6). As confirmation, high H^2^ values were calculated for the two traits (0.97 for RS and 0.99 for AAC). Together with the continuous distribution of the phenotypic data related to RS and AAC, this makes our dataset particularly relevant for studying the genetic bases of the two starch traits in *japonica* rice through a GWAS approach. A lower genetic effect was instead recorded for TS (H^2^ = 0.33).

To assess relationships among RS evaluations in brown non-cooked and brown cooked kernels, twenty genotypes, randomly selected among the low, medium, and high RS classes, were subjected to cooking tests to evaluate the RS content after the cooking process. The range of variability was from 0.026 to 0.241% and from 0.185 to 1.223% in not cooked and cooked samples, respectively (Supplementary Table S7). Results revealed that RS in not cooked and in cooked rice was highly correlated (*R* = 0.805, *p* ≤ 0.001), confirming overall, that the determined hierarchy based on RS content in uncooked rice was also maintained after cooking.

### 2.2. Genotypic Analysis of the Japonica Rice Population

The panel of 115 rice accessions was genotyped by GBS. After pruning, 28,445 SNPs were utilized for the GWA mapping of RS, AAC, and TS-related regions. The SNP panel was enriched by the addition of the following two SNPs identified within the *Wx* gene [1]: TBGI270314, localized in the *Wx* intron 1, and TBGI270316, lying inside the *Wx* exon 6. Based on the rice genome size of 373 Mb [40], the average density of the filtered markers along the genome was 13.12 Kbp/marker (ranging from 7.72 Kbp/marker for chromosome 10 to 20.86 Kbp/marker for chromosome 3; Supplementary Table S8).

**Figure 4:**
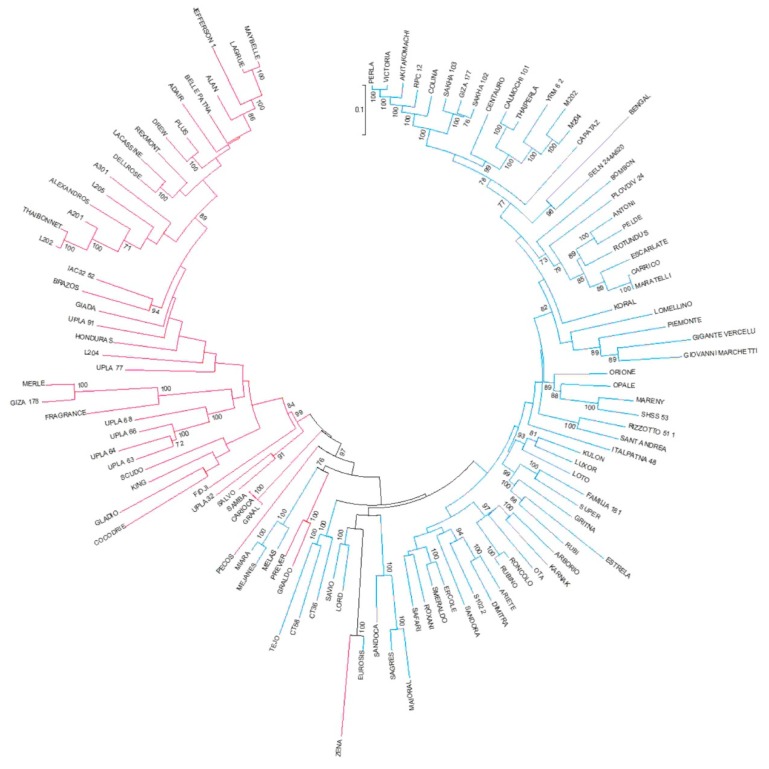
Neighbor-joining tree of the rice panel used in the present study. The two colors indicate the two different taxonomic groups, blue = temperate *japonica*; red = tropical *japonica*.

The clustering analysis utilized for mining the population structure subdivided the 115 genotypes into two clusters according to the temperate and tropical *japonica* subgroups (Figure 4).

The same separation between tropical and temperate groups was obtained by the first component (PC1) of a PCA analysis, which accounted for 24.31% of total genetic variability (Supplementary Figure S2). The second component (PC2) accounted for 7.08% of variability and separated a small subgroup of tropical *japonica* accessions represented by Scudo, Prever, Carioca, Graldo, Samba, and Graal (Supplementary Figure S2). All these genotypes are classified as Long B (Supplementary Table S1) and come from Italy, excluding Graal, which is from France. The separation of these accessions from the total panel might suggest a common origin.

A mean LD decay of 1,247 kb (ranging from 415 kb for chromosome 11 to 1,845 for chromosome 4) was calculated over the physical distance in the whole panel of 115 accessions (Supplementary Figure S3).

### 2.3. Genome Wide Association Analysis for RS, AAC and TS

A GWAS experiment was conducted to identify the loci associated with the variation in AAC, RS, and TS content among the 115 rice accessions.

No associated regions were identified for TS, likely because of the low genetic variability related to this trait in our genetic material.

The list of all the RS- or AAC-associated SNPs is reported in Supplementary Table S9, while Table 1 summarizes the main information for each RS- or AAC-associated region.

**Figure 5:**
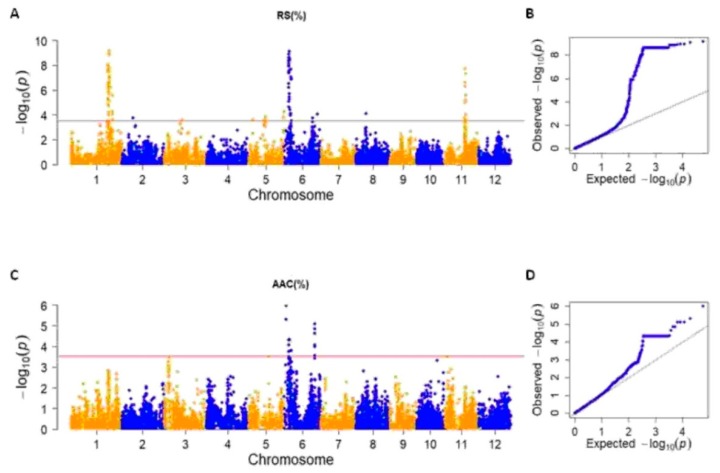
Manhattan plots and Q-Q plots of the significant associations detected for resistant starch (RS; (**A**,**B**)) and apparent amylose content (AAC; (**C**,**D**)).

A total of 11 significant RS-related marker-trait associations (RS_MTAs) were discovered on chromosomes 1, 2, 3, 5, 6, 8, and 11 (Figure 5A,B; Table 1 and Supplementary Table S9), with −log10(p) values (referred to the peak markers) ranging between 3.59 to 9.15 and explained phenotypic variance (R^2^) from 11% to 29% (Figure 5A,B; Table 1 and Supplementary Table S9).

Only three RS_MTAs were defined by single SNPs, as follows: RS_MTA2, related to SNP S2_9401106 on chromosome 2; RS_MTA3, defined by S3_15491784 on chromosome 3; and RS_MTA6_2, corresponding to S6_24395396 on chromosome 6 (Figure 5A; Table 1 and Supplementary Table S9). The other RS_MTAs were defined by two or more SNPs co-segregating with the QTL peaks (Figure 5A; Table 1 and Supplementary Table S9).

The most significantly RS-associated MTA was the chromosome 1 RS_MTA1 (−log10(p) = 9.15 and R^2^ = 0.29), defined by 167 significant SNPs (peak marker S1_32380039), located from 31,035,257 to 35,233,919 bp, and belonging to a LD block from 30,645,440 to 33,485,305 bp (Figure 5A; Table 1 and Supplementary Table S9).

Chromosomes 5 and 6 contained the highest number of RS-related MTAs (3 RS_MTAs each; Figure 5A; Table 1 and Supplementary Table S9). In particular, chromosome 6 included the second most significantly RS-associated region (RS_MTA6_1), related to S6_4418603 (-log10(p) = 9.10), spanning a 2,042,006 bp region (from 4,160,208 to 6,202,214 bp) defined by 121 SNPs and included in a LD block from 4,160,208 to 4,930,358 bp (Figure 5A; Table 1 and Supplementary Table S9).

For AAC, 5 MTAs were discovered on chromosome 6 (−log10(p) from 5.99 to 3.90 and R^2^ from 0.12 to 0.19; Figure 5C,D; Table 1 and Supplementary Table S9). The most significant associations were recorded for the two SNPs located on the *Wx* gene, the main determinant of AAC [4], as follows: TBGI270314 (AAC_MTA6_1, –log10(p) = 5.30 and R^2^ = 0.17) and TBGI270316 (AAC_MTA6_2, –log10(p) = 5.99 and R^2^ = 0.19; Figure 5C; Table 1 and Supplementary Table S9).

The analysis of the specific alleles associated with the two *Wx* SNPs revealed the presence of three haplotypes in our rice collection, as follows: GA (34 accessions; the first letter indicates the haplotype for SNP TBGI270314, while the second letter corresponds to SNP TBGI270316), GC (28 accessions), and TA (53 accessions; Supplementary Tables S1 and S10). According to previous analyses [1], the AAC values of the GA and GC accessions were significantly different with respect to the ones recorded for the TA accessions that showed AAC values lower than 19.50% (Supplementary Tables S1, S10 and S11). Slightly different AAC values were also observed between the GA and GC accessions, with GA associated with AAC levels from 16.03 to 19.95% and higher than 22.31%, while GC mainly associated with AAC ranging from 20.65 to 24.65% (Supplementary Tables S1, S10 and S11).

The correlation between the haplotypes at the *Wx* intron 1 SNP (TBGI270314) and grain shape was previously observed [1]. In our analysis, the grain shape-related traits of the genotypes showing the *Wx^a^* (G) allele were significantly different from those recorded for the *Wx^b^* (T) accessions, particularly for SWSL that was higher for *Wx^a^* genotypes, in comparison to the *Wx^b^* ones (Supplementary Tables S1, S12 and S13), as previously reported [1].

Considering the other AAC-MTAs discovered, interestingly, the local LD decay analysis showed that the LD blocks calculated for AAC_MTA6_3 and AAC_MTA6_4 (from 4,160,208 to 4,930,358 bp and from 5,593,250 to 6,222,678 bp, respectively) were included in the one recorded for RS_MTA6_1 (Table 1).

### 2.4. Identification of Candidate Genes

**Figure 6:**
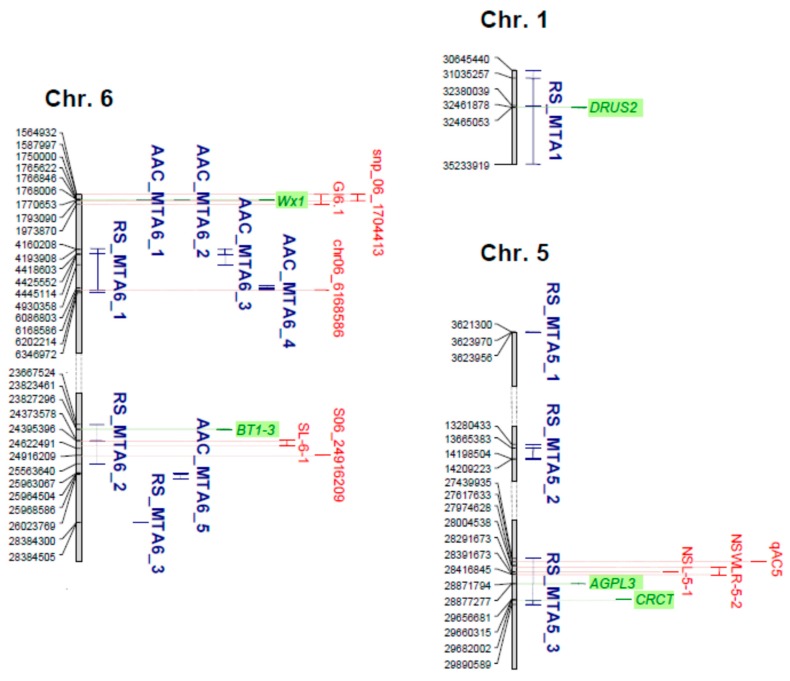
Distribution of the significant associations detected on chromosomes 1, 5, and 6 (**blue**). The known genes (**green**) or quantitative trait loci (QTLs; **red**) related to starch or grain shape parameters that co-localize with the detected marker-trait associations (MTAs) are reported.

To find out candidate genes affecting RS and/or AAC levels, a search on the Oryzabase database (https://shigen.nig.ac.jp/rice/oryzabase/) was conducted for the genes annotated as implicated in starch metabolism and in the determination of amylose content present in and located at the level or in proximity (±1.20 kbp) of the RS- and/or AAC-related MTAs and their LD blocks. The analysis was enriched by literature searching. Results are summarized in Figure 6 and Table 2.

Among the candidate loci affecting RS content, *DRUS2* (Dwarf and Runtish Spikelet 2; Os01g0769700) was located within the RS_MTA1 associated region on chromosome 1 (Figure 6, Table 1; Table 2). Two genes on chromosome 5 were associated to RS_MTA5_3, as follows: Os05g0580000, encoding for AGPL3 (ADP-Glucose Pyrophosphorylase Large subunit 3), a regulatory enzyme for plant starch and amylose synthesis, was located inside the RS_MTA5_3 LD block, and the CRCT (CO2-Responsive CONSTANS, CONSTANS-like, and Time of Chlorophyll a/b Binding Protein1 (CCT) Protein) encoding locus Os05g0595300 was proximal to RS_MTA5_3 (Figure 6, Table 1; Table 2). As last, *BT1-3* (*Brittle1-3*; Os06g0602700), encoding for an adenylate translocator which facilitates the transfer of extraplastidial synthesized ADPglucose into amyloplasts in maize [41], was included in the LD block calculated for RS_MTA6_2 (Figure 6, Table 1; Table 2). No candidate genes were discovered for the other RS_MTAs and for AAC_MTA6_3, AAC_MTA6_4, or AAC_MTA6_5 (Table 1; Table 2), suggesting that they represent new associations to RS and/or AAC levels.

A searching for previously mapped QTLs involved in the determination of starch properties and co-localized with the RS- and/or AAC-related MTAs detected in the present work revealed a positional correspondence between RS_MTA6_1 and AAC_MTA6_4 and the RS-associated locus chr06_6168586 detected by Bao et al. [24] (Figure 6). The *qAC5* QTL, affecting the amylose content in rice [42], was found to be located in the LD block recorded for RS_MTA5_3 (Figure 6). Significant AAC-associated SNPs were recently discovered on chromosome 6 through a GWAS conducted on 320 *indica* rice genotypes and, among these, a SNP in the promoter region of the *Wx* gene contributed to haplotypes discriminating samples into different amylose classes [43]. In addition, the chromosome 6 GI6.1 region, identified by integrating a GWAS and a TWAS (transcriptome-wide association study) on a panel of 305 *indica* varieties, represents a major hot spot associated to the rice glycaemic index (GI), involving 26 genes including *Wx* [44]. Both these MTAs showed a co-positional relationship with the two most significant MTAs for AAC (AAC_MTA6_1 and AAC_MTA6_2) identified in the present work (Figure 6). As confirmation of the correlations between RS and the grain shape-related traits, two RS-related MTAs overlapped respectively with two QTLs associated with grain shape and identified in previous GWAS analyses using larger rice collections, including the genotypes utilized in the present work [45,46] (Figure 6). The first one was represented by RS_MTA_5_3, which overlapped with the chromosome 5 QTLs NSL5-1, associated with the naked seed length, and NSWLR5-2, related to the naked seed width/length ratio [46] (Figure 6). The second one was RS_MTA6_2, located inside the seed length-associated locus SL6-1 on chromosome 6 [46]. Furthermore, the RS_MTA6_2 LD block included the seed length/seed width ratio (SR)-associated region, defined by the S6_24916209 SNP and detected by Biscarini et al. [45] (Figure 6).

## 3. Discussion

### 3.1. Correlation Between Grain Quality Related Traits

High variability was recorded for RS values among the 115 rice accessions considered, even though the average RS measured in our work was lower than the one detected for the *japonica* genotypes utilized by Bao et al. [24] (average RS = 0.45%). This difference might be related to the different genetic materials utilized and to the different method used for RS quantification, which has been shown to result in variable RS values [22,32]. Most of the evaluations are, in fact, carried out on boiled flour obtained from polished grains, which is known to increase RS values, while our evaluations were based on non-cooked brown kernels. Indeed, when RS was evaluated on cooked brown rice of 20 random accessions from our panel, recorded RS ranged from 0.185 to 1.223%, with an average value of 0.54%, which is in agreement with evaluations of Bao et al. [24]. Our evaluations on brown uncooked rice are in the range of RS values detected for *japonica* rice using the same procedure [21].

Additional agreements with previous works [24,25,35] were found for the correlation between RS and AAC. This result further confirms the implication of AAC in RS determination, as already suggested [24].

Biselli et al. [1] observed that, in rice, AAC correlated with SW and grain length/grain width ratio (abbreviated as LW by authors). Genotypes with low AAC (<21.5%) showed low LW values, while accessions having intermediate or high AAC (>21.5%) displayed high LW values [1]. This behavior has been explained as the result of a “correlational selection”, defined as the selection of optimal character combinations, generating genetic correlation between suites of linked traits [47]. Thus, long and thin-grain rice is used for cooking applications like rice salads, pilafs, garnishes, rice casserole dishes, and as accompaniment to sauces requiring a distinct shape and a firm texture, which is ensured by high AAC; while short- and medium-grain rice genotypes, showing low AAC, are proper for cooking applications which require soft, moist, and sticky rice grains, like the preparation of puddings and desserts [1]. According to these results, in the present work, AAC correlated positively with SL and NSL, while negative correlations were observed between AAC and SW, NSW, SWSL, and NSWNSL. The same type of correlation discovered for AAC and the grain shape-related traits was calculated for RS (and RSTS), suggesting that correlational selection also occurred for RS, together with AAC and grain shape. However, the associations between RS and grain shape-related traits might only be a secondary result of the high association between AAC and RS, and so the human selection for grain shape and AAC could have indirectly determined the occurrence of low RS cultivars with bold grains and high RS genotypes with slender grains.

The distribution of RS showed no relationship with geographic origin, like what was observed for barley [48]. On the other hand, the mean RS, RSTS, and AAC values of the temperate *japonica* genotypes belonging to our collection were significantly lower, with respect to those calculated for the tropical accessions. The same situation was found in the research conducted by Bao et al. [24], for which the utilized temperate *japonica* accessions showed the lowest levels of RS and AAC. Differences between temperate and tropical *japonica* accessions were also discovered for the grain shape related traits, according to the fact that tropical *japonica* rice grains are usually longer and thinner with respect to the temperate ones [49]. These differences suggest that the correlational selection for AAC and grain shape and, consequently, RS, had possibly marked the phenotypic distinction between tropical and temperate *japonica* according to human preferences for grain characteristics.

### 3.2. GWAS Analyses for RS and AAC and Identification of Candidate Genes

The rice population utilized in this work represents a sub-set of the panel used by Volante et al. [46], which was demonstrated to be suitable for GWAS studies. The mean LD decay calculated for our rice panel (1.247 kbp) was higher with respect to those present in literature [50]. This discrepancy has been previously explained as being related to factors like SNP densities and/or kinship among accessions [45], and, in our case, the low population size. However, similar or even higher LD values in rice populations were previously highlighted [51,52].

In the present work, a total of 11 significant associations with RS were observed on chromosomes 1, 2, 3 5, 6, 8, and 11, while the GWAS analysis performed for AAC led to the identification of 5 significant associations on chromosome 6. According to the fact that RS and AAC positively correlate and that AAC was hypothesized to be the main determinant of RS [24], the two AAC-MTAs, AAC_MTA6_2 and AAC_MTA6_4, coincided with RS_MTA6_1.

The analysis of genes implicated in starch metabolism and located at the level or in proximity of the RS-associated QTLs led to the identification of three candidate genes affecting the RS content. The following two of them were positioned inside RS_MTA5_3: The *AGPL3* locus Os05g0580000 and Os05g0595300, encoding for CRCT (CO2-Responsive CONSTANS, CONSTANS-like, and Time of Chlorophyll a/b Binding Protein1 (CCT) Protein). AGPase, which catalyzes the first rate-limiting reaction of starch biosynthesis, was demonstrated to have an active role in RS determination by the fact that, as mentioned above, high RS rice mutants showed lower activities of SBE and AGPase and higher activities of SS and DBE [35]. CRCT was suggested as a positive regulator of starch accumulation in vegetative tissues because of its effect on the expression of starch synthesis genes in response to the levels of photoassimilates [53]. In fact, it was observed that the expression of several genes implicated in starch biosynthesis, like AGPase, positively correlated with the *CRCT* expression and the starch content in the leaf sheath increased in *CRCT* overexpression lines while they decreased in knockdown lines [53]. Thus, *CRCT* might indirectly affect the RS content because of its effect on starch synthesis genes.

*OsBT1-3* (*BRITTLE 1-3*; Os06g0602700; from 23,823,461 to 23,827,296 bp on chromosome 6) was located at the level of the RS_MTA6_2 LD block. The *BRITTLE1* gene encodes for an adenylate translocator, which facilitates the transfer of extraplastidial synthesized ADPglucose, the substrate of SSs and GBSSI, into amyloplasts in maize [41]. Mutations of maize *BRITTLE1* caused a reduction in the amount of starch produced in the endosperm [54,55]. Thus, allelic variation at the *OsBT1-3* locus might reduce the ADPglucose available for SSs in amyloplasts, with alteration of the amylose/amylopectin ratio and consequently changing in the RS amount. On these bases, *OsBT1-3* might represent a genuine candidate for RS determination.

The *DRUS2* (Dwarf and Runtish Spikelet 2; Os01g0769700) gene, encoding a receptor-like kinase [56], was found inside the RS_MTA1 QTL. Despite the fact that there is no evidence of a direct implication of this gene in starch metabolism, it was demonstrated that *DRUS2*, together with its closely related gene *DRUS1*, affects sugar utilization during rice reproductive development, probably by stimulating the expression of the gene encoding for the UGP2 (UDP-glucose pyrophosphorylase 2) enzyme, which catalyzes the interconversion between starch and sucrose metabolites [57], and thus starch biosynthesis in pollen [56]. By this view, an indirect effect of *DRUS2* on starch metabolism and RS can be also postulated on rice seeds.

As expected, the most significant association with AAC was discovered for the SNPs located at the level of intron 1 (AAC_MTA6_1, detected by TBGI270314) and exon 6 (AAC_MTA6_2, detected by TBGI270316), respectively, of the *Wx* gene, the main determinant of AAC [1,5,6,7]. In agreement with our results, a significant association between amylose content (AC) and SNPs on *Wx* was also found in a recent GWAS study on rice eating and cooking characteristics (AC, gel consistency, and gelatinization temperature), using 174 and 84 *indica* and *japonica* rice genotypes, respectively [42]. This analysis also identified a QTL associated with AC, *qAC5*, which was located in the LD block recorded for RS_MTA5_3 [42], thus supporting the involvement of RS_MTA5_3 in RS determination. Moreover, a SNP in the promoter region of the *Wx* gene contributing to haplotypes related to different amylose classes [43] and a major hot spot associated with the rice glycaemic index (GI) that included *Wx* [44] were both co-localized with AAC_MTA6_1 and AAC_MTA_2, thus further underlying the importance of *Wx* in determining rice grain healthy properties.

An additional positional correspondence with known starch-related QTLs present in literature was observed between RS_MTA6_1 and AAC_MTA6_4, which co-localized with the RS-associated putative locus chr06_6168586, detected in Bao et al. [24], further confirming the association between the two MTAs identified and starch-related parameters. Moreover, the phenotypic variance explained by RS_MTA6_1 (29%) was higher with respect to the one calculated for chr06_6168586 (9.5%) [24], probably because of the different genotypes involved in the two analyses.

In a previous research aimed at improving the marker-based predictability of AAC, the two *Wx* SNPs utilized in the present study were demonstrated to be reliable tools for AAC prediction in breeding programs [1]. Three different haplotypes for the combination of the two SNPs were associated with specific AAC values, as follows: TA corresponded to AAC from waxy to 22%, GC was present in accessions with 22–24% AAC, and GA was found in accessions with AAC > 24% [1]. According to these data, the same haplotypes were discovered in the current research and showed a similar association with AAC values, with some exception probably related to the different genotypes utilized, as follows: TA was associated with AAC < 19.5%, GA corresponded to AAC from 16.03 to 19.95% and >22.31%, and GC was associated to AAC from 20.65 to 24.65%.

In addition, the *Wx* intron 1 SNP (TBGI270314) was strongly associated with SW and LW as *Wx^a^* genotypes (G allele) showed thinner grains with a higher length/width ratio, with respect to *Wx^b^* (T allele) [1]. Similar results were recorded for the present germplasm collection, for which the SWSL and NSWNSL values were lower for the *Wx^a^* genotypes with respect to the ones recorded for the accessions carrying the T allele. All these data represent a further confirmation of the hypothesis that correlational selection for grain shape and AAC potently marked the evolution of rice according to customer’s requests [1].

As previously indicated, in the present work, significant correlations were recorded between RS and grain shape-related traits. These observations were enforced by the discovery of correspondences between two of the RS_MTAs and QTLs associated with grain shape-related traits mapped in previous research, as follows: RS_MTA5_3 co-localized with the NSL5-1 and NSWLR5-2 QTLs, associated with naked seed length and the ratio between naked seed width/length, respectively [46]; RS_MTA6_2 co-localized with the seed length-related QTL SL6-1 detected by Volante et al. [46]; while the RS_MTA6_2 LD block included the region associated with the seed length/width ratio related to the peak marker S6_24916209 [45].

No candidate genes or co-localizations with known starch-related QTLs were discovered for RS_MTA2, RS_MTA3, RS_MTA5_2, RS_MTA6_3, RS_MTA8, and RS_MTA11 or for AAC_MTA6_5, indicating that they could represent new QTLs, contributing to the variation of grain starch-related traits, and can be considered new sources for RS or AAC determination.

## 4. Materials and Methods

### 4.1. Plant Materials

The rice population utilized in this work consisted of 115 accessions and represents a sub-group of a previously described rice germplasm panel [45,46,58] (Supplementary Table S1). The population included 70 temperate and 45 tropical *japonica* genotypes well adapted to European agro-climatic conditions, even though they were bred in different countries, as follows: A total of 40 accessions in Italy, 25 in the USA, 8 in Argentina, 11 in Portugal, and the remaining in other locations (Supplementary Table S1). The Italian varieties were selected because of their relevance for breeding programs and their large contribution to rice production in the last decades, while the other genotypes were either largely used in Italian rice breeding or represent reference varieties at the international level. These accessions belong to the Rice Germplasm collection maintained at the CREA-Research Centre for Cereal and Industrial Crops (Vercelli, Italy). Plants were grown in field trials using standard agronomic practices. Briefly, rice accessions were grown in 2013 in replicated field plots at the CREA-Research Centre for Cereal and Industrial Crops, Vercelli (45°19’21.96” N, 8°22’24.07” E) in Eastern Piedmont, Italy. The climate is characterized by summer mean annual temperatures of ~23 °C and an average annual precipitation 1300 mm. The soils are classified as silty clay loams (sand 47.8%, loam 42.8%, clay 9.41%) with pH = 6.36. Plots were fertilized pre-sowing in April with a dry organic fertilizer, “Verdazoto” (260 kg/ha 12.5% N); top-dress fertilization was then added at the end of June (300 kg/ha, 20-0-30). Sowing was performed in dry conditions. The plots were flooded (10 cm water) when most of the accessions reached the three-leaf stage (typically after 30 days) and kept in this condition throughout the whole growing season until 30 days before harvesting. The collected seeds were used for the phenotypic analyses described in the next section.

### 4.2. Phenotypic Analyses

De-hulled kernels were used for the phenotypic analyses and are referred to as ‘seeds’ throughout the paper. Seed moisture was measured just before the chemical analyses; 1 g of brown rice was dried in an oven at 130 °C until reaching a stable weight (120 min). All analytical measurements were performed in two replicates and, because of the predominant genetic influence on starch properties [59], they were performed considering one year and one environment, as usual for this kind of data [24]. Total starch (TS) content was determined according to the AOAC (2005) Official Method 996.11, using the Total Starch Assay Kit (Megazyme, Ireland), on 100 mg of milled sample (particle size < 0.5 mm). Briefly, the GOPOD (Glucose oxidase/peroxidase) reagent was added to 100 μL of digested sample in the reading of the absorbance at 510 nm by a Lambda Bio20 spectrophotometer (Perkin Elmer, Italy). A solution of D-glucose (100 μL of 1 mg/ml D-glucose standard solution) was used as the control. Data were expressed as weight percentage (w/w) on a dry weight basis (% dw).

RS content was determined on 100 mg of milled sample (particle size < 1 mm) according to the AOAC (2005) Official Method 2002.02, using the Resistant Starch Assay Kit (Megazyme, Ireland). After the hydrolysis with KOH 2M at 4 °C, RS was measured by adding the GOPOD reagent to 100 μL of the sample and following the procedure adopted for TS, described above.

The cooking test was performed following the procedure of Ranghino [60], slightly modified to adapt the procedure to brown rice. Briefly, 50 g of rice kernels were added to 650 mL of boiling tap water and a cooking time of 26 min was applied. Cooked rice was immediately frozen, lyophilized, and stored at −20 °C until the analyses.

AAC values used in the present work were determined in a previous study on the same materials here used, where the adopted procedures for AAC determination are also detailed [1].

The traits related to seed morphology used here were recorded on the same materials in a previous work [46], in which 100 hulled and naked seeds of each accession (with three biological replicates) were analyzed using a scanner-based image analysis through the software WinSeedlePro v.2011. The considered grain shape-related traits were the following: Hulled seed length (SL), naked seed length (NSL), hulled seed width (SW), naked seed width (NSW), ratio between SW and SL (SWSL), and ratio between NSW and NSL (NSWNSL).

### 4.3. Statistical Analyses of Phenotypic Data

Frequency distributions of phenotypic data were tested for normality using the Shapiro–Wilk test in R environment. Two-factor ANOVA was carried out using JMP v.8 software (SAS Institute, 2008) to evaluate differences for TS, RS, and AAC between genotypes. Broad sense heritability (H^2^) was estimated from the variance components, obtained by fitting both replications and genotypes as random terms as H^2^ = σ^2^g/(σ^2^g + σ^2^e), where σ^2^g is the genotypic variance component and σ^2^e is the residual variance component.

To find correlation among traits, Pearson’s coefficients were calculated in R using the standard “cor.test” function and the significance of correlations was assessed by the t-test implemented in the “cor.test” function.

The Wilcoxon rank sum test, performed by the JMP v.8 software (SAS Institute, 2008), was utilized to compare the mean values of the traits related to starch or grain shape among the different RS clusters, the temperate and tropical *japonica* genotypes, the different AAC classes, and the cultivars carrying different *Wx* alleles.

Hierarchical clustering was used to create relatively homogenous groups of rice genotypes using two variables, RS and the ratio between RS and TS (RSTS), as classification criteria. The cluster analysis, based on Euclidean distances, was carried out by the JMP v.8 software (SAS Institute, 2008).

### 4.4. Genotyping

All the accessions were genotyped by sequencing according to a pipeline described previously [46]. The initial SNP dataset, containing 246,026 SNPs mapped on the Os-Nipponbare Reference-IRGSP-1.0 pseudomolecule assembly (https://rapdb.dna.affrc.go.jp/download/irgsp1.html), was filtered using the PLINK program [61]. Markers with a call rate lower than 95% and a MAF (minimal allele frequency) lower than 5% were discarded. After filtering, a total of 28,445 SNPs were used for the GWAS analysis.

Two additional SNPs, associated with the *Wx* gene (TBGI270314, located on *Wx* intron 1 at 1,766,846 bp, and TBGI270316, located on *Wx* exon 6 at 1,768,006 bp), were added, for a total of 28,447 SNP markers. The genotyping of the two SNPs was performed by direct sequencing as described by Biselli et al. [1].

### 4.5. Population Structure and Linkage Disequilibrium Decay

A random subset of 9996 SNPs (833 markers/chromosome) was utilized for the analysis of genetic stratification in the rice panel. Two different methods were adopted, as follows: A principal component analysis (PCA) performed by the Tassel v5.2.0 software [62] and a phylogenetic clustering obtained utilizing MEGA7 software [63] with the neighbour-joining method and the Jukes–Cantor genetic distance.

The linkage disequilibrium (LD; r^2^) analysis was performed, as described by Volante et al. [46], using 5000 randomly selected SNPs and the R package “LDcorSV v1.3.1” [64], with the structure membership matrix as a covariate. The values were averaged in 10 kb windows for each rice chromosome. A mean value was obtained for each distance class. The resulting values were plotted against distance and fitted to a LOESS curve by an R script [65,66]. The critical value for r^2^ between unlinked loci was set as 0.2. The physical distance corresponding to a LOESS value of 0.2 was assumed as the extent of LD.

Haploview v4.2 software [67], with default settings, was utilized for calculating local LD around the detected MTAs.

### 4.6. Association Mapping

GWAS analyses were performed for the three starch-related traits, RS, AAC, and TS, according to Volante et al. [46]. Tassel v5.2.0 software [62] was used for the analysis. The following parameters were selected: No compression, genetic and residual variance estimated for each marker (P3D OFF). The mixed linear model (MLM) with the kinship matrix (K), as a random effect for taking into account for population stratification, was utilized. The BIC (Bayesian information criterion) was calculated in order to find the best method for each trait. For each SNP marker, a *p*-value of the association to each trait was calculated. The threshold to declare a marker as associated was set to 0.05 after correction for multiple testing using the false discovering rate (FDR) method [68]. The R package “qqman” (https://cran.r-project.org/web/packages/qqman/index.html) was utilized to draw Manhattan plots and Q-Q plots for each trait. Single-SNP associations were considered true positive when a peak of multiple SNPs was detected at lower −log10 (*p*-values) in the Manhattan plot. SNP clusters in full LD with the same *p*-value were considered as peak regions. The regions associated to each trait were aligned with the results of the Haploview analysis described above.

### 4.7. Identification of Candidate Genes Affecting RS and/or AAC

For each trait, the regions ranging from −120 kb to +120 kb around each associated marker (corresponding to an average LD decay of 0.5 estimated on the LOESS curve) were screened for candidate genes. The identification of candidate genes for each trait was performed by selecting the genes annotated in the Oryzabase database (https://shigen.nig.ac.jp/rice/oryzabase/), considering the gene onthologies “starch metabolism” and “amylose content”, and genes described in literature to affect RS or AAC, located at the level of or in proximity to the MTAs detected.

## 5. Conclusions

Considerable phenotypic variation for three grain quality-related traits, represented by RS, AAC, and TS, was present in the analyzed *japonica* rice collection. A significant positive correlation was found between AAC and RS, according to the observation that AAC represents one of the main determinants of RS in rice seeds [24], and between these two starch-related traits and grain shape. Correlational selection of AAC and grain shape has been inferred previously [1]. The association recorded between RS and grain shape-related traits also suggests that RS might be subjected to correlational selection, even if, due to the positive correlation between RS and AAC, these associations could be only an indirect result of the selection for AAC and grain size. Moreover, the different values for AAC, RS, and the grain shape parameters displayed by the tropical and temperate *japonica* accessions belonging to our rice collection indicate that the correlational selection for AAC and grain shape, and maybe RS, had probably marked the different grain characteristic between tropical and temperate *japonica* cultivars according to human preferences.

The GWAS analyses conducted for the starch-related parameters considered in this study identified 11 and 5 MTAs related to RS and AAC, respectively. Two AAC-associated MTAs corresponded to one RS_MTA, thus highlighting the correlation between AAC and RS. Candidate genes affecting starch properties were located at the level of or in proximity to five of the identified starch-related MTAs, thus providing a list of genes that can be exploited to dissect the genetic bases of the MTAs. The new candidate genes and the new RS- and/or AAC-associated regions detected provide valuable sources for future functional characterization of starch-related genetic determinants and for breeding programs aimed at improving rice grain quality.

## Figures and Tables

**Table 1:** Associations between single nucleotide polymorphism (SNP) markers and resistant starch (RS) or apparent amylose content (AAC). For each trait, the related marker-trait associations (MTAs), with the corresponding peak marker/region (SNPs with the highest *p*-value) are reported. Multiple markers with the same *p*-value are reported as a peak region. For each peak marker/markers, the associated chromosome (Chr), the *p*-value (expressed like -log10(*p*)), the number of trait-associated SNPs located in its related region, the start and end positions and the size of the related region, the variance explained (R^2^), and the start and end positions of the related LD block are reported.

Trait	MTA	Peak Marker/Region (bp)	Chr	-Log10(p)	SNPs	Associated Region (bp)			Peak Marker R^2^	LD Block (bp)
						Start	End	Size		
RS	RS_MTA1	S1_32380039	1	9.15	167	31,035,257	35,233,919	4,198,662	0.29	30,645,440–33,485,305
	RS_MTA2	S2_9401106	2	3.75	1	9,401,106	-	-	0.12	-
	RS_MTA3	S3_15491784	3	3.60	1	15,491,784	-	-	0.11	-
	RS_MTA5_1	S5_3623956-S5_3623970	5	3.59	2	3,623,956	3,623,970	14	0.11	-
	RS_MTA5_2	S5_14198504	5	3.81	14	13,665,383	14,209,223	543,840	0.12	13,280,433–14,210,256
	RS_MTA5_3	S5_29890589	5	4.24	2	29,682,002	29,890,589	208,587	0.13	27,439,935–29,890,589
	RS_MTA6_1	S6_4418603	6	9.10	121	4,160,208	6,202,214	2,042,006	0.29	4,160,208–6,346,972
	RS_MTA6_2	S6_24395396	6	3.74	1	24,395,396	-	-	0.12	23,667,524–25,563,640
	RS_MTA6_3	S6_28384300-S6_28384505	6	4.08	2	28,384,300	28,384,505	205	0.13	-
	RS_MTA8	S8_8352009-S8_8367922	8	4.10	2	8,352,009	8,367,922	15,913	0.13	8,313,616–8,332,478; 8,422,453–8,441,645
	RS_MTA11	S11_18059435	11	7.73	13	17,462,389	18,145,389	683,000	0.25	17,418,693–18,592,536
AAC	AAC_MTA6_1	TBGI270314	6	5.30	1	1,766,846	-	-	0.17	-
	AAC_MTA6_2	TBGI270316	6	5.99	1	1,768,006	-	-	0.19	-
	AAC_MTA6_3	S6_4425552	6	4.35	76	4,193,908	4,445,114	251,206	0.14	4,160,208–4,930,358
	AAC_MTA6_4	S6_6086803-S6_6202214	6	3.80	5	6,086,803	6,202,214	115,411	0.12	5,593,250–6,222,678
	AAC_MTA6_5	S6_25964504-S6_25968586	6	5.10	9	25,963,067	26,023,769	60,702	0.16	25,963,067–27,324,453

**Table 2:** Summary of the candidate genes identified for resistant starch (RS)- and/or apparent amylose content (AAC)-related marker-trait associations (MTAs). For each gene, the related trait, gene description, specific chromosome (Chr), RAPD ID, position on the Nipponbare reference genome (https://rapdb.dna.affrc.go.jp/), and the RS- and/or AAC-MTA/MTAs are reported.

Trait	Gene	Gene Description	Chr	RAP ID	Position (bp)		MTAs
					Start	End	
RS	*DRUS2*	Dwarf and Runtish Spikelet 2	1	Os01g0769700	32,461,878	32,465,053	RS_MTA1
RS	*AGPL3*	ADPGlucose Pyrophosphorylase Large subunit 3	5	Os05g0580000	28,871,794	28,877,277	RS_MTA5_3
RS	*CRCT*	CO2-Responsive CONSTANS, CONSTANS-like, and Time of Chlorophyll a/b Binding Protein1 (CCT)	5	Os05g0595300	29,656,681	29,660,315	RS_MTA5_3
AAC	*Wx1*, *GBSSI*	Waxy. Granule Bound Starch Synthase I	6	Os06g0133000	1,765,622	1,770,653	AAC_MTA6_1, AAC_MTA6_2
RS	*BT1-3*	Brittle1-3	6	Os06g0602700	23,823,461	23,827,296	RS_MTA6_2

## Supplementary Materials

The following are available online at https://www.mdpi.com/2223-7747/8/8/292/s1, Supplemental Tables S1 and S9 (Supplementary Tables.xlsx), Supplementary Tables S2–S6 and S8–S13 (Supplementary Tables.docx), Supplementary Figures S1–S3 (Supplementary Figures.) are provided. Figure S1: Correlation between TS or RSTS and grain shape-related traits., Figure S2: Principal component analysis (PCA) of the rice panel used in the present study, Figure S3: Analysis of the mean LD decay, Table S1: List of the 115 rice genotypes utilized in the present study, Table S2: Results of the variance analyses, performed by the Wilcoxon Rank Sum test, conducted to compare the mean values of each phenotypic trait between RS clusters. Table S3: Summary of the mean values and the ranges of variation for each grain shape-related trait recorded for each AAC class, Table S4: Results of the variance analyses, performed by the Wilcoxon Rank Sum test, conducted to compare the mean values of each grain shape-related trait of each AAC class, Table S5: Summary of the mean values and the ranges of variation for each phenotypic trait recorded for the 70 temperate and 45 tropical japonica rice genotypes considered in the present study and results of the variance analyses, performed by the Wilcoxon Rank Sum test, conducted to compare the mean values of each phenotypic trait of each japonica group, Table S6: ANOVA results for RS (resistant starch) and AAC (Apparent amylose content), Table S7: RS (resistant starch) content after the cooking process of a random sample of the accessions utilized in the present study, Table S8: Distribution of the SNPs on rice chromosomes, Table S9: List of all the RS- and/or AAC-associated SNPs identified in the GWAS analyses, Table S10: Summary of the mean values and the ranges of variation of AAC (apparent amylose content) for each haplotype related to the two SNP located on the *Wx* gene, Table S11: Results of the variance analyses, performed by the Wilcoxon Rank Sum test, conducted to compare the AAC (apparent amylose content) mean values of each haplotype related to the two SNP located on the *Wx* gene, Table S12: Summary of the mean values and the ranges of grain shape-related traits for each haplotype related to the *Wx* intron 1 SNP TBGI270314, Table S13: Results of the variance analyses, performed by the Wilcoxon Rank Sum test, conducted to compare the grain shape-related trait mean values of each haplotype related to the *Wx* intron 1 SNP TBGI270314.

## Abbreviations

AAC	apparent amylose content
AGPase	ADP-glucose pyrosphorylase
DBE	starch debranching enzyme
GBS	genotyping by sequencing
GBSSI	granule bound starch synthase I
GWAS	Genome Wide Association Scan
MTA	marker-trait association
QTL	quantitative trait locus
RS	resistant starch
SBE	starch branching enzyme
SS	starch synthase
TS	total starch
*Wx*	*Waxy*
