# Supplementary material for: GWAS for Starch-Related Parameters in Japonica Rice (Oryza sativa L.)
Source: Plants (Basel). 2019 Aug 19;8(8):292. doi: 10.3390/plants8080292 (PMC6724095; doi:10.3390/plants8080292)
Supplement: Supplementary file 1 [file plants-08-00292-s001.zip › plants-528719-suppl-final/Figure S1.pdf]

A

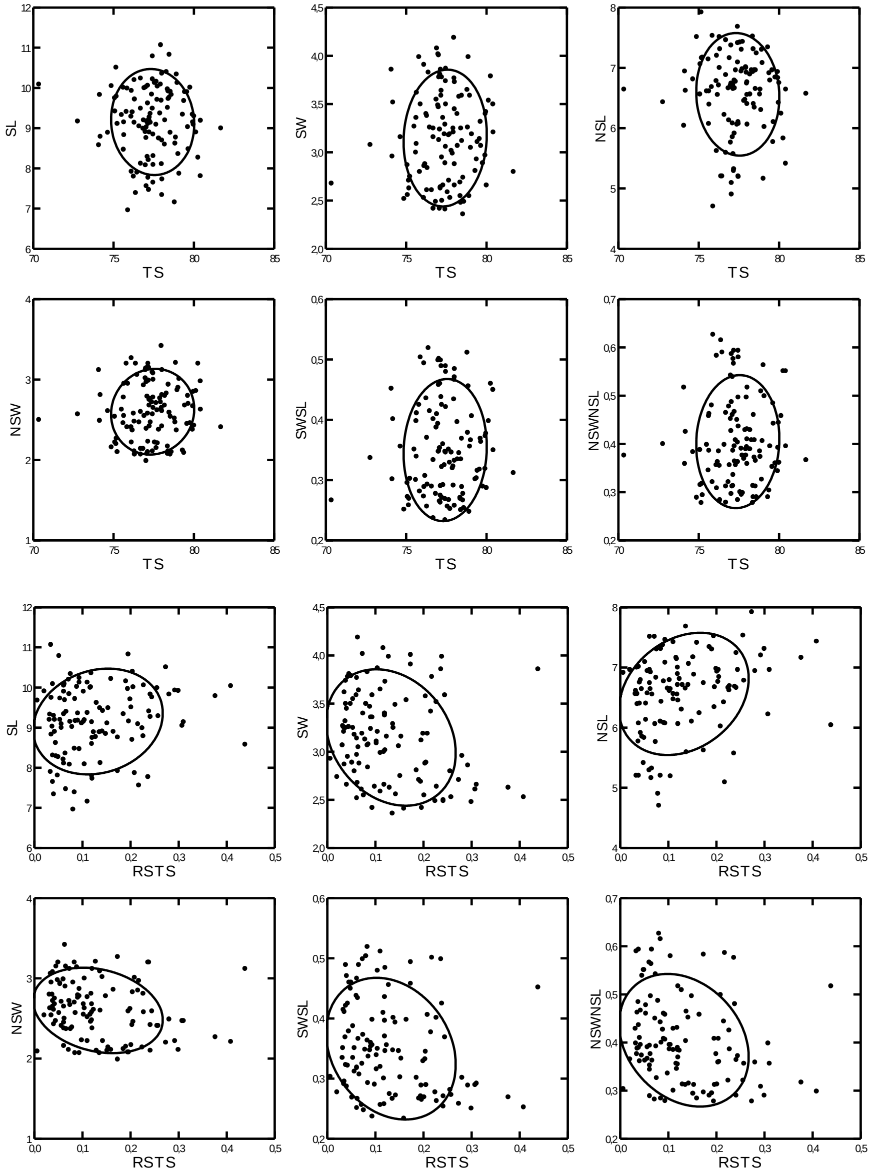

B

Trait 1	Trait 2	R	p-value
TS	SL	-0.044	0.641
TS	NSL	-0.044	0.637
TS	SW	0.055	0.563
TS	NSW	0.060	0.523
TS	SWSL	0.049	0.603
TS	NSWNSL	0.046	0.623
RSTS	SL	0.156	0.095
RSTS	NSL	0.252	0.007
RSTS	SW	-0.228	0.014
RSTS	NSW	-0.225	0.016
RSTS	SWSL	-0.222	0.017
RSTS	NSWNSL	-0.251	0.007

Figure S1. Correlation between TS or RSTS and grain shape-related traits. **A** Scatterplots of TS or RSTS and grain shape-related traits, representing the bivariate distribution for each pair of traits (Trait 1 and Trait 2). Confidence ellipses mark confidence limit for each distribution (P = 0.95). **B** Pearson's correlation coefficients (R) for each pair of traits. P-values are also reported. TS = total starch; RSTS = ration between resistant starch (RS) and TS; SL = seed length; SW = seed width; NSL = naked seed length; NSW = naked seed width; SWSL = ratio between SW and SL; NSWNSL = ratio between NSW and NSL.
