# Supplementary material for: GWAS for Starch-Related Parameters in Japonica Rice (Oryza sativa L.)
Source: Plants (Basel). 2019 Aug 19;8(8):292. doi: 10.3390/plants8080292 (PMC6724095; doi:10.3390/plants8080292)
Supplement: Supplementary file 1 [file plants-08-00292-s001.zip › plants-528719-suppl-final/Figure S2.pdf]

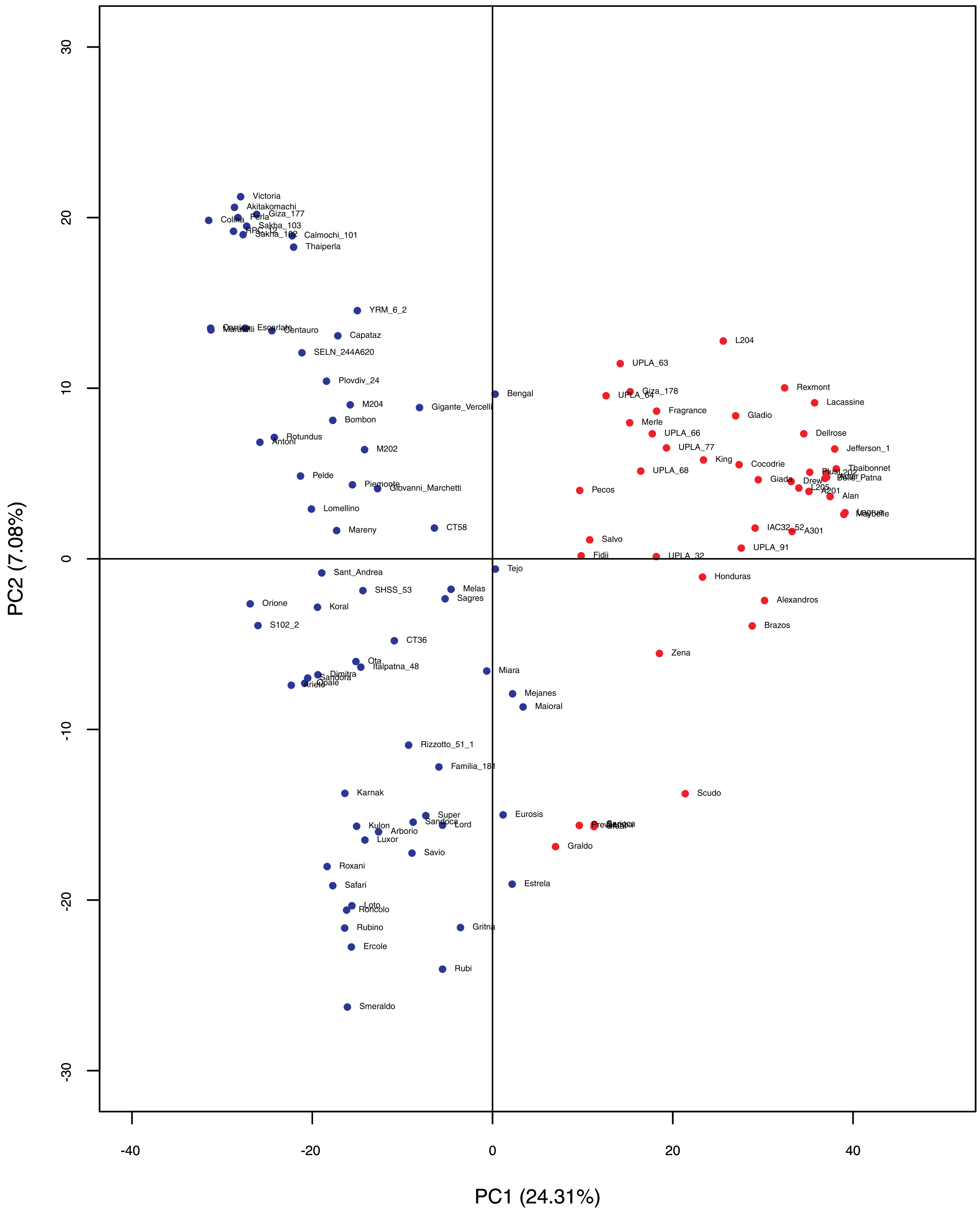

Figure S2. Principal component analysis (PCA) of the rice panel used in the present study. Point colours correspond to the two different taxonomic groups: blue = temperate *japonica*; red = tropical *japonica*.
