# Supplementary material for: GWAS for Starch-Related Parameters in Japonica Rice (Oryza sativa L.)
Source: Plants (Basel). 2019 Aug 19;8(8):292. doi: 10.3390/plants8080292 (PMC6724095; doi:10.3390/plants8080292)
Supplement: Supplementary file 1 [file plants-08-00292-s001.zip › plants-528719-suppl-final/Figure S3.pdf]

A

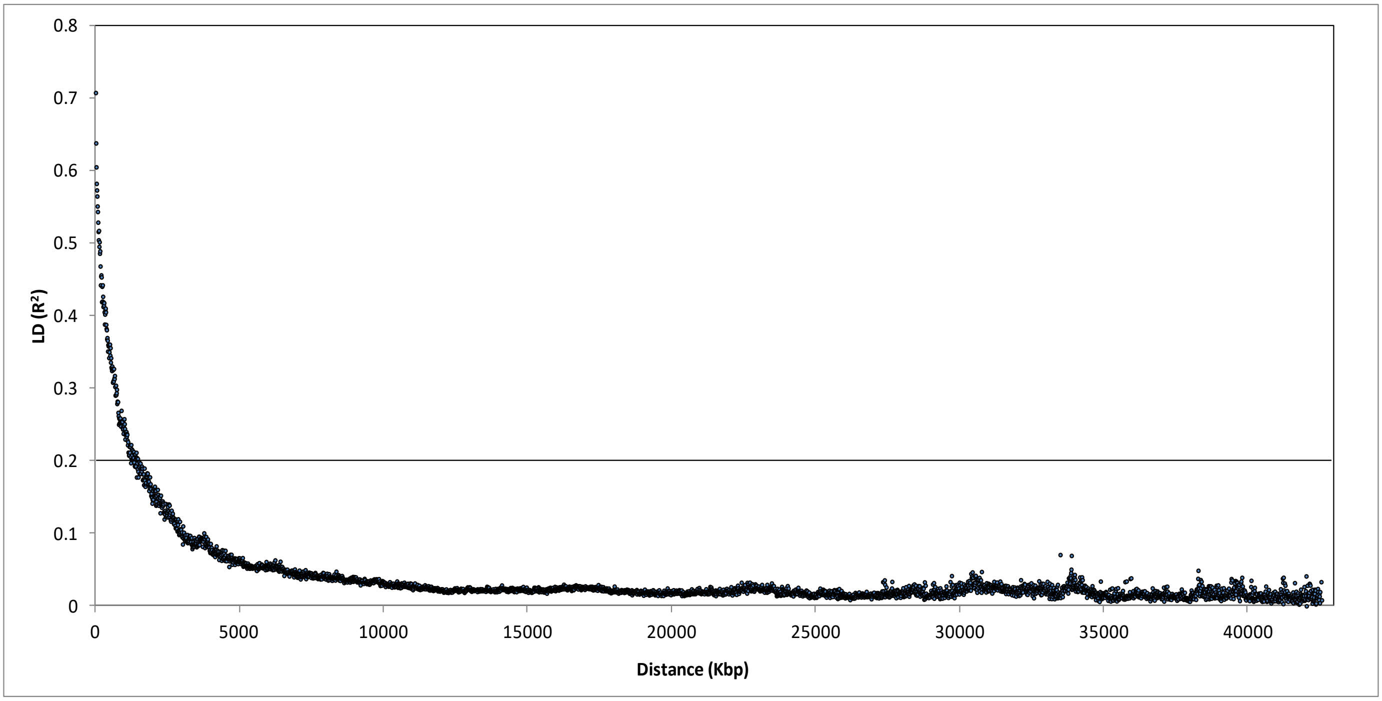

B

Chromosome	LD decay (kbp)
1	765
2	875
3	1,315
4	1,845
5	1,545
6	845
7	1,555
8	1,585
9	975
10	1,835
11	415
12	1,405
avg	1,247

Figure S3. Analysis of the mean LD decay. **A** Mean LD (R^2) as a function of marker physical distance in the panel of the 115 rice accessions utilized in this study. The horizontal red line shows the critical R^2 level between linked markers. **B** Summary of the mean LD decay calculated for each chromosome.
