# Supplementary material for: GWAS for Starch-Related Parameters in Japonica Rice (Oryza sativa L.)
Source: Plants (Basel). 2019 Aug 19;8(8):292. doi: 10.3390/plants8080292 (PMC6724095; doi:10.3390/plants8080292)
Supplement: Supplementary file 1 [file plants-08-00292-s001.zip › plants-528719-suppl-final/Table S10.docx]

**Table S10.** Summary of the mean values and the ranges of variation of AAC (apparent amylose content) for each haplotype related to the two single nucleotide polymorphisms (SNPs) located on the *Waxy* gene. The first letter indicates the haplotype for the intron 1 SNP TBGI270314, whereas the second letter refers to the exon 6 SNP TBGI270316. The number of accessions for each haplotype is reported. SD = standard deviation.

| ***Wx* haplotype** | **n.** | **AAC**  **Mean ± SD (%)** | **AAC**  **Range of variation (%)** |
| --- | --- | --- | --- |
| GA | 34 | 20.75 ± 2.93 | 16.03 – 25.16 |
| GC | 28 | 22.91 ± 2.80 | 19.47 – 25.21 |
| TA | 53 | 18.15 ± 2.60 | 3.47 – 19.35 |
