# Supplementary material for: GWAS for Starch-Related Parameters in Japonica Rice (Oryza sativa L.)
Source: Plants (Basel). 2019 Aug 19;8(8):292. doi: 10.3390/plants8080292 (PMC6724095; doi:10.3390/plants8080292)
Supplement: Supplementary file 1 [file plants-08-00292-s001.zip › plants-528719-suppl-final/Table S11.docx]

**Table S11.** Results of the variance analyses, performed by the Wilcoxon Rank Sum test, conducted to compare the AAC (apparent amylose content) mean values of each haplotype related to the two single nucleotide polymorphisms (SNPs) located on the *Waxy* gene. The first letter indicates the haplotype for the intron 1 SNP TBGI270314, whereas the second letter refers to the exon 6 SNP TBGI270316.

| ***Wx* haplotypes** | | **Z** | **P-value** |
| --- | --- | --- | --- |
| GA | GC | 1.96 | 0.05 |
| GA | TA | -2.62 | 0.009 |
| GC | TA | -4.57 | <0.001 |
