# Supplementary material for: GWAS for Starch-Related Parameters in Japonica Rice (Oryza sativa L.)
Source: Plants (Basel). 2019 Aug 19;8(8):292. doi: 10.3390/plants8080292 (PMC6724095; doi:10.3390/plants8080292)
Supplement: Supplementary file 1 [file plants-08-00292-s001.zip › plants-528719-suppl-final/Table S12.docx]

**Table S12.** Summary of the mean values and the ranges of grain shape-related traits for each haplotype related to the *Waxy* intron 1 single nucleotide polymorphism (SNP) TBGI270314. The number of accessions for each haplotype is reported. SD = standard deviation; SL = seed length; SW = seed width; NSL = naked seed length; NSW = naked seed width; SWSL = ratio between SW and SL; NSWNSL = ratio between NSW and NSL.

| **TBGI270314 haplotype** | **n.** | **Trait** | **Mean ± SD** | **Range of variation** |
| --- | --- | --- | --- | --- |
| G | 62 | SL (mm) | 9.38 ± 0.74 | 7.54 – 11.05 |
|  |  | SW (mm) | 3.05 ± 0.47 | 2.35 – 4.07 |
|  |  | NSL (mm) | 6.73 ± 0.60 | 5.08 – 7.91 |
|  |  | NSW (mm) | 2.53 ± 0.35 | 1.98 – 3.19 |
|  |  | SWSL | 0.33 ± 0.069 | 0.233 – 0.500 |
|  |  | NSWNSL | 0.38 ± 0.083 | 0.282 – 0.585 |
| T | 53 | SL (mm) | 8.90 ± 0.92 | 6.94 – 10.77 |
|  |  | SW (mm) | 3.25 ± 0.44 | 2.41 – 4.18 |
|  |  | NSL (mm) | 6.38 ± 0.69 | 4.69 – 7.50 |
|  |  | NSW (mm) | 2.67 ± 0.34 | 2.06 – 3.41 |
|  |  | SWSL | 0.37 ± 0.080 | 0.236 – 0.518 |
|  |  | NSWNSL | 0.43 ± 0.092 | 0.277 – 0.613 |
