# Supplementary material for: GWAS for Starch-Related Parameters in Japonica Rice (Oryza sativa L.)
Source: Plants (Basel). 2019 Aug 19;8(8):292. doi: 10.3390/plants8080292 (PMC6724095; doi:10.3390/plants8080292)
Supplement: Supplementary file 1 [file plants-08-00292-s001.zip › plants-528719-suppl-final/Table S13.docx]

**Table S13.** Results of the variance analyses, performed by the Wilcoxon Rank Sum test, conducted to compare the grain shape-related trait mean values of each haplotype related to the *Waxy* intron 1 single nucleotide polymorphism (SNP) TBGI270314. SL = seed length; SW = seed width; NSL = naked seed length; NSW = naked seed width; SWSL = ratio between SW and SL; NSWNSL = ratio between NSW and NSL.

| **Grain shape-related traits and TBGI270314** **haplotype** | | **Z** | **P-value** |
| --- | --- | --- | --- |
| SL_G | SL_T | -2.65 | 0.008 |
| SW_G | SW_T | 2.58 | 0.010 |
| NSL_G | NSL_T | -2.63 | 0.009 |
| NSW_G | NSW_T | 2.35 | 0.019 |
| SWSL_G | SWSL_T | 2.50 | 0.012 |
| NSWNSL_G | NSWNSL_T | 2.66 | 0.008 |
