# Supplementary material for: GWAS for Starch-Related Parameters in Japonica Rice (Oryza sativa L.)
Source: Plants (Basel). 2019 Aug 19;8(8):292. doi: 10.3390/plants8080292 (PMC6724095; doi:10.3390/plants8080292)
Supplement: Supplementary file 1 [file plants-08-00292-s001.zip › plants-528719-suppl-final/Table S2.docx]

**Table S2.** Results of the variance analyses, performed by the Wilcoxon Rank Sum test, conducted to compare the mean values of each phenotypic trait between resistant starch (RS) clusters. RS = resistant starch; TS = total starch; RSTS = ratio between RS and TS; AAC = apparent amylose content; SL = seed length; SW = seed width; NSL = naked seed length; NSW = naked seed width; SWSL = ratio between SW and SL; NSWNSL = ratio between NSW and NSL.

| **Trait** | **Contrast of RS clusters** | | **Z** | **P-value** |
| --- | --- | --- | --- | --- |
| TS | 1 | 2 | -1.81 | 0.238 |
|  | 1 | 3 | -0.580 | 0.562 |
|  | 2 | 3 | 0.330 | 0.741 |
| RS | 1 | 2 | 4.458 | <0.001 |
|  | 1 | 3 | 4.937 | <0.001 |
|  | 2 | 3 | 4.458 | <0.001 |
| RS/TS | 1 | 2 | 4.458 | <0.001 |
|  | 1 | 3 | 4.937 | <0.001 |
|  | 2 | 3 | 4.457 | <0.001 |
| AAC | 1 | 2 | 1.968 | 0.049 |
|  | 1 | 3 | 4.170 | <0.001 |
|  | 2 | 3 | 2.527 | 0.012 |
| SL | 1 | 2 | -3.34 | 0.001 |
|  | 1 | 3 | -0.009 | 0.993 |
|  | 2 | 3 | 3.149 | 0.002 |
| NSL | 1 | 2 | -2.273 | 0.023 |
|  | 1 | 3 | 0.010 | 0.992 |
|  | 2 | 3 | 3.027 | 0.002 |
| SW | 1 | 2 | 2.337 | 0.019 |
|  | 1 | 3 | -0.655 | 0.513 |
|  | 2 | 3 | -3.533 | <0.001 |
| NSW | 1 | 2 | 2.337 | 0.019 |
|  | 1 | 3 | -1.09 | 0.276 |
|  | 2 | 3 | -3.647 | <0.001 |
| SW/SL | 1 | 2 | 3.01 | 0.003 |
|  | 1 | 3 | -0.075 | 0.94 |
|  | 2 | 3 | -3.873 | <0.001 |
| NSW/NSL | 1 | 2 | 3.086 | 0.002 |
|  | 1 | 3 | -0.785 | 0.432 |
|  | 2 | 3 | -4.458 | <0.001 |
