# Supplementary material for: GWAS for Starch-Related Parameters in Japonica Rice (Oryza sativa L.)
Source: Plants (Basel). 2019 Aug 19;8(8):292. doi: 10.3390/plants8080292 (PMC6724095; doi:10.3390/plants8080292)
Supplement: Supplementary file 1 [file plants-08-00292-s001.zip › plants-528719-suppl-final/Table S3.docx]

**Table S3.** Summary of the mean values and the ranges of variation for each grain shape-related trait recorded for each apparent amylose content (AAC) class. The number of accessions for each AAC class is reported. SD = standard deviation; AAC = apparent amylose content; SL = seed length; SW = seed width; NSL = naked seed length; NSW = naked seed width; SWSL = ratio between SW and SL; NSWNSL = ratio between NSW and NSL.

| **AAC class** | **N. of accessions** | **Grain shape-related trait** | **Mean ± SD** | **Range of variation** |
| --- | --- | --- | --- | --- |
| low | 67 | SL (mm) | 8.97 ± 0.96 | 6.94 – 11.05 |
|  |  | NSL (mm) | 6.36 ± 0.71 | 4.69 – 7.67 |
|  |  | SW (mm) | 3.28 ± 0.39 | 2.41 – 4.18 |
|  |  | NSW (mm) | 2.70 ± 0.33 | 2.06 – 3.41 |
|  |  | SWSL | 0.37 ± 0.076 | 0.235 – 0.518 |
|  |  | NSWNSL | 0.43 ± 0.093 | 0.277 – 0.625 |
| medium | 46 | SL (mm) | 9.40 ± 0.64 | 7.54 – 10.49 |
|  |  | NSL (mm) | 6.82 ± 0.47 | 5.08 – 7.91 |
|  |  | SW (mm) | 2.96 ± 0.51 | 2.35 – 4.07 |
|  |  | NSW (mm) | 2.47 ± 0.33 | 1.98 – 3.20 |
|  |  | SWSL | 0.32 ± 0.067 | 0.233 – 0.500 |
|  |  | NSWNSL | 0.37 ± 0.069 | 0.276 – 0.585 |
| high | 2 | SL (mm) | 9.65 ± 0.46 | 9.32 – 9.97 |
|  |  | NSL (mm) | 7.43 ± 0.13 | 7.33 – 7.52 |
|  |  | SW (mm) | 2.80 ± 0.007 | 2.79 – 2.80 |
|  |  | NSW (mm) | 2.26 ± 0.21 | 2.11 – 2.40 |
|  |  | SWSL | 0.29 ± 0.014 | 0.280 – 0.300 |
|  |  | NSWNSL | 0.37 ± 0.022 | 0.288 – 0.319 |
