# Supplementary material for: GWAS for Starch-Related Parameters in Japonica Rice (Oryza sativa L.)
Source: Plants (Basel). 2019 Aug 19;8(8):292. doi: 10.3390/plants8080292 (PMC6724095; doi:10.3390/plants8080292)
Supplement: Supplementary file 1 [file plants-08-00292-s001.zip › plants-528719-suppl-final/Table S4.docx]

**Table S4.** Results of the variance analyses, performed by the Wilcoxon Rank Sum test, conducted to compare the mean values of each grain shape-related trait of each apparent amylose content (AAC) class. AAC = apparent amylose content; SL = seed length; SW = seed width; NSL = naked seed length; NSW = naked seed width; SWSL = ratio between SW and SL; NSWNSL = ratio between NSW and NSL.

| **Grain shape-related trait** | **AAC classes** | | **Z** | **p-value** |
| --- | --- | --- | --- | --- |
| SL | low | high | 1.342 | 0.180 |
|  | medium | high | -0.447 | 0.655 |
|  | medium | low | 2.305 | 0.021 |
| NSL | low | high | 0.447 | 0.655 |
|  | medium | high | -0.475 | 0.655 |
|  | medium | low | 3.458 | 0.001 |
| SW | low | high | -1.342 | 0.180 |
|  | medium | high | -1.342 | 0.180 |
|  | medium | low | -3.076 | 0.002 |
| NSW | low | high | -1.342 | 0.180 |
|  | medium | high | -0.447 | 0.655 |
|  | medium | low | -3.290 | 0.001 |
| SWSL | low | high | -1.342 | 0.180 |
|  | medium | high | -0.447 | 0.655 |
|  | medium | low | -3.567 | <0.001 |
| NSWNSL | low | high | -1.342 | 0.180 |
|  | medium | high | 0.447 | 0.655 |
|  | medium | low | -3.884 | <0.001 |
