# Supplementary material for: GWAS for Starch-Related Parameters in Japonica Rice (Oryza sativa L.)
Source: Plants (Basel). 2019 Aug 19;8(8):292. doi: 10.3390/plants8080292 (PMC6724095; doi:10.3390/plants8080292)
Supplement: Supplementary file 1 [file plants-08-00292-s001.zip › plants-528719-suppl-final/Table S5.docx]

**Table S5.** Summary of the mean values and the ranges of variation for each phenotypic trait recorded for the 70 temperate and 45 tropical *japonica* rice genotypes considered in the present study and results of the variance analyses, performed by the Wilcoxon Rank Sum test, conducted to compare the mean values of each phenotypic trait of each *japonica* group. SD = standard deviation; RS = resistant starch; TS = total starch; RSTS = ratio between RS and TS; AAC = apparent amylose content; SL = seed length; SW = seed width; NSL = naked seed length; NSW = naked seed width; SWSL = ratio between SW and SL; NSWNSL = ratio between NSW and NSL.

| **Trait** | | **Temperate** | **Tropical** | **Z** | **P-value** |
| --- | --- | --- | --- | --- | --- |
| TS (%) | Mean ± SD | 77.48 ± 1.58 | 77.35 ± 1.87 | -0.96 | 0.34 |
|  | Range of variation | 72.82 – 80.47 | 70.40 – 81.73 |  |  |
| RS (%) | Mean ± SD | 0.080 ± 0.054 | 0.137 ± 0.071 | 5.84 | <0.001 |
|  | Range of variation | 0.006 – 0.326 | 0.028 – 0.319 |  |  |
| RSTS (%) | Mean ± SD | 0.103 ± 0.071 | 0.178 ± 0.093 | 5.84 | <0.001 |
|  | Range of variation | 0.008 – 0.440 | 0.036 – 0.410 |  |  |
| AAC (%) | Mean ± SD | 18.86 ± 2.84 | 21.97 ± 2.63 | 5.50 | <0.001 |
|  | Range of variation | 3.47 – 24.85 | 14.92 – 25.21 |  |  |
| SL (mm) | Mean ± SD | 8.87 ± 0.89 | 9.6 ± 0.60 | 3.92 | <0.001 |
|  | Range of variation | 6.94 – 10.81 | 7.85 – 11.05 |  |  |
| SW (mm) | Mean ± SD | 3.40 ± 0.36 | 2.80 ± 0.31 | -5.41 | <0.001 |
|  | Range of variation | 2.58 – 4.18 | 2.35 – 4.00 |  |  |
| NSL (mm) | Mean ± SD | 6.34 ± 0.66 | 6.90 ± 0.51 | 4.13 | <0.001 |
|  | Range of variation | 4.69 – 7.67 | 4.89 – 7.91 |  |  |
| NSW (mm) | Mean ± SD | 2.78 ± 0.28 | 2.31 ± 0.22 | -5.53 | <0.001 |
|  | Range of variation | 2.06 – 3.41 | 1.98 – 3.00 |  |  |
| SWSL | Mean ± SD | 0.39 ± 0.068 | 0.29 ± 0.043 | -5.39 | <0.001 |
|  | Range of variation | 0.255 – 0.518 | 0.233 – 0.457 |  |  |
| NSWNSL | Mean ± SD | 0.45 ± 0.083 | 0.34 ± 0.053 | -5.20 | <0.001 |
|  | Range of variation | 0.282 – 0.625 | 0.276 – 0.498 |  |  |
