# Supplementary material for: GWAS for Starch-Related Parameters in Japonica Rice (Oryza sativa L.)
Source: Plants (Basel). 2019 Aug 19;8(8):292. doi: 10.3390/plants8080292 (PMC6724095; doi:10.3390/plants8080292)
Supplement: Supplementary file 1 [file plants-08-00292-s001.zip › plants-528719-suppl-final/Table S6.docx]

**Table S6.** ANOVA results for RS (resistant starch) and AAC (Apparent amylose content).

| **Trait** | **Source** | **DF** | **Sum of Squares** | **Mean Square** | **F Ratio** | **Prob > F** |
| --- | --- | --- | --- | --- | --- | --- |
| RS | Genotype | 114 | 0,93 | 0,0081 | 53,42 | 7,18E-58 |
|  | Error | 96 | 0,015 | 0,00015 | - | - |
|  | C. total | 210 | 0,94 | - | - | - |
| AAC | Genotype | 114 | 2789.23 | 35.31 | 481.21 | 1.14E-159 |
|  | Error | 160 | 11.74 | 0.073 | - | - |
|  | C. total | 239 | 2800.96 | - | - | - |
