# Supplementary material for: GWAS for Starch-Related Parameters in Japonica Rice (Oryza sativa L.)
Source: Plants (Basel). 2019 Aug 19;8(8):292. doi: 10.3390/plants8080292 (PMC6724095; doi:10.3390/plants8080292)
Supplement: Supplementary file 1 [file plants-08-00292-s001.zip › plants-528719-suppl-final/Table S7.docx]

**Table S7.** RS (resistant starch) content after the cooking process of a random sample of the accessions utilized in the present study. Pearson’s correlation coefficients (R), with the relative p-value, between RS in not cooked grains and RS in cooked grains is reported.

|  | **RS (%)** | |
| --- | --- | --- |
| **Genotypes** | **Not cooked** | **Cooked** |
| Smeraldo | 0.026 | 0.233 |
| Opale | 0.026 | 0.301 |
| Seln 244 A6-20 | 0.027 | 0.295 |
| Luxor | 0.028 | 0.255 |
| Prever | 0.059 | 0.185 |
| Scudo | 0.06 | 0.405 |
| Gritna | 0.071 | 0.348 |
| Ercole | 0.071 | 0.336 |
| Maioral | 0.075 | 0.540 |
| Zena | 0.078 | 0.266 |
| Dimitra | 0.079 | 0.383 |
| Orione | 0.081 | 0.279 |
| Dellrose | 0.098 | 0.869 |
| Capataz | 0.102 | 0.680 |
| Sakha 102 | 0.107 | 0.470 |
| Gigante Vercelli | 0.11 | 1.203 |
| Gladio | 0.197 | 0.798 |
| Upla 63 | 0.207 | 0.960 |
| Thaibonnet | 0.236 | 0.891 |
| Merle' | 0.241 | 1.223 |
| **Mean** | 0.099 ± 0.068 | 0.546 ± 0.331 |
| **R^2^** | 0.805 (p ≤ 0.001) | |
