# Supplementary material for: GWAS for Starch-Related Parameters in Japonica Rice (Oryza sativa L.)
Source: Plants (Basel). 2019 Aug 19;8(8):292. doi: 10.3390/plants8080292 (PMC6724095; doi:10.3390/plants8080292)
Supplement: Supplementary file 1 [file plants-08-00292-s001.zip › plants-528719-suppl-final/Table S8.docx]

|  |
| --- |

**Table S8.** Distribution of the single nucleotide polymorphisms (SNPs) on rice chromosomes. For each chromosome (Chr) the number of total SNPs (SNPs tot) detected, the estimated (estim.) length and the estimated SNP density are reported.

| **Chr** | **SNPs tot** | **Estim. length (kbp)^1^** | **Estim. marker density (kbp/SNP)** |
| --- | --- | --- | --- |
| 1 | 3,265 | 43,270.923 | 13.25 |
| 2 | 2,135 | 35,937.25 | 16.83 |
| 3 | 1,746 | 36,413.819 | 20.86 |
| 4 | 2,555 | 35,502.694 | 13.90 |
| 5 | 1,937 | 29,958.434 | 15.47 |
| 6 | 2,080 | 31,248.787 | 15.02 |
| 7 | 2,251 | 29,697.621 | 13.19 |
| 8 | 2,568 | 28,443.022 | 11.08 |
| 9 | 1,358 | 23,012.72 | 16.95 |
| 10 | 3,007 | 23,207.287 | 7.72 |
| 11 | 3,392 | 29,021.106 | 8.56 |
| 12 | 2,153 | 27,531.856 | 12.79 |
| All | 28,447 | 373,245.519 | 13.12 |
| ^1^ according to Kawahara *et al.* [40]. | | |  |
